# Bone in heart failure

**DOI:** 10.1002/jcsm.12516

**Published:** 2020-02-22

**Authors:** Goran Loncar, Natasa Cvetinovic, Mitja Lainscak, Andjelka Isaković, Stephan von Haehling

**Affiliations:** ^1^ Institute for Cardiovascular Diseases Dedinje Belgrade Serbia; ^2^ Faculty of Medicine University of Belgrade Belgrade Serbia; ^3^ Department of Cardiology and Pneumology University Medical Center Goettingen, Georg‐August University Goettingen Germany; ^4^ Department of Cardiology University Clinical Hospital Center ‘Dr. Dragisa Misovic‐Dedinje’ Belgrade Serbia; ^5^ Department of Internal Medicine General Hospital Murska Sobota Murska Sobota Slovenia; ^6^ Faculty of Medicine University of Ljubljana Ljubljana Slovenia; ^7^ DZHK (German Centre for Cardiovascular Research), partner site Goettingen Goettingen Germany

**Keywords:** Heart failure, Osteoporosis, Fractures, Bone mineral density, Markers of bone metabolism

## Abstract

There is an increasing interest in osteoporosis and reduced bone mineral density affecting not only post‐menopausal women but also men, particularly with coexisting chronic diseases. Bone status in patients with stable chronic heart failure (HF) has been rarely studied so far. HF and osteoporosis are highly prevalent aging‐related syndromes that exact a huge impact on society. Both disorders are common causes of loss of function and independence, and of prolonged hospitalizations, presenting a heavy burden on the health care system. The most devastating complication of osteoporosis is hip fracture, which is associated with high mortality risk and among those who survive, leads to a loss of function and independence often necessitating admission to long‐term care. Current HF guidelines do not suggest screening methods or patient education in terms of osteoporosis or osteoporotic fracture. This review may serve as a solid base to discuss the need for bone health evaluation in HF patients.

## Heart failure

Heart failure (HF) is a major public health problem affecting millions of patients worldwide. The overall prevalence of HF is increasing, because of the aging of the population, the success in prolonging survival in patients suffering coronary events, and the success in postponing coronary events by effective prevention in those at high risk or those who have already survived a first event. The prevalence of HF is approximately 1–2% of the adult population in developed countries, rising to ≥10% among people over 70 years of age.^1^ The outcome of patients with HF is poor. The most recent European data demonstrate that 12 month all‐cause mortality rates for acute HF and stable/ambulatory HF patients were 24% and 6%, respectively.^2^ In Europe, there is an increasing burden of hospitalizations due to HF.^3^, ^4^, ^5^ Indeed, HF is a clinical syndrome associated with diverse metabolic disturbances, many of which may adversely influence musculoskeletal and fat metabolism and provoke weight loss, that is, exaggerated loss of all body compartments (bone, skeletal muscle, and fat tissue) that may finally lead to cachexia.^6^, ^7^, ^8^

## Heart failure and body wasting

The phenomenon of involuntary weight loss in chronic disease has been known for centuries.^9^ Cachexia in HF can be diagnosed and defined as involuntary non‐oedematous weight loss ≥6% of total body weight within the previous 6–12 months^10^, ^11^; however, several definitions have been used in clinical studies. Substantial weight loss is a strong indicator of imminent death in the course of the disease.^12^, ^13^ It is also assumed that weight loss is not the cause of death but a strong predictor of poor prognosis. In addition, cachexia in HF, otherwise known as cardiac cachexia, is associated not only with poor outcomes but also with an unfavourable response to drug treatment and poor quality of life.^14^, ^15^ The causes are multifactorial, and in individual patients, they are difficult to determine. These may include pro‐inflammatory immune activation, neurohormonal derangements, poor nutrition and malabsorption, impaired calorie and protein balance, anabolic hormone resistance, reduced anabolic drive, and prolonged immobilization and physical deconditioning, together characterized as catabolic/anabolic imbalance.^16^ For the first time, the importance of the body wasting in HF has been recently outlined in the separate paragraph in the guidelines on the management of HF established by the European Society of Cardiology.^17^ However, osteoporosis has only been mentioned, without highlighting its importance for serious complications in these patients (such as hip fractures) that can lead to invalidity and death, particularly in those patients who are clinically frail.^18^, ^19^ Both osteoporosis and HF may induce and potentiate each other as we would like to evaluate it in the following paragraphs. The literature on the relationship between HF and bone status was reviewed by searching relevant PubMed references (keywords: heart failure, bone loss, osteoporosis, osteopenia, fractures).

## Osteoporosis and risk for heart failure

Disorders of bone metabolism, among which osteoporosis is the most prominent, are characteristics of physiological aging and commonly coexist with chronic disease, having adverse influence on quality of life. In parallel, osteoporosis has been suggested as an independent risk factor for cardiovascular disease.^20^ Low bone mineral density (BMD) is a risk factor for increased mortality in later life, especially from cardiovascular disease.^21^ Common underlying biological processes might contribute to vascular calcification and bone demineralization.^22^ Additionally, low BMD predicts incident HF in healthy individuals.^23^ Recently, another study has added to existing evidence linking low BMD with a higher rate of incident HF specifically for white men, while calling into question a similar association for white women.^24^ Additionally, decreased BMD was independently associated with left ventricular (LV) diastolic dysfunction.^25^, ^26^ The mechanisms between reduced BMD and LV diastolic dysfunction remain unclear. One potential reason is that calcification of the arterial tissue resembles the process of osteogenesis, leading to impaired ventricle–vessel coupling due to arterial stiffness.^27^, ^28^ Increased arterial stiffness raises LV afterload by elevating systolic blood pressure.^29^ Several reports support the hypothesis of an interplay between reduced BMD, elevated arterial stiffness, and LV diastolic dysfunction.^30^, ^31^

## Heart failure and the risk of osteoporosis and osteoporotic fractures

There is an increasing interest in osteoporosis and reduced BMD affecting not only post‐menopausal women but also men, particularly those with coexisting chronic diseases. Bone status in patients with stable chronic HF has been rarely studied so far (*Table* 1).^32^, ^33^, ^34^ In total, available data suggest that stable pre‐transplant chronic HF patients have a significant but only moderately disturbed bone metabolism compared with cachectic HF patients or those who are candidates for heart transplantation.^35^ HF and osteoporosis are highly prevalent aging‐related syndromes that exact an enormous impact on society. Both disorders are common causes of loss of function and independence, and of prolonged hospitalizations, presenting a heavy burden to the health care system. It remains an unanswered question whether HF leads to osteoporosis and frailty fractures, or if it is just an epiphenomenon as a passive participant in a population at risk for both syndromes. We have previously shown that bone mineral content (BMC) and BMD were reduced in non‐cachectic men with HF, unlike to total lean and fat mass.^32^ Jankowska *et al*. confirmed reduced BMD and BMC in men with HF, which were predicted by HF severity and anabolic depletion.^33^ Although it can be expected that during HF progression changes in different tissue compartments are related, Jankowska *et al*. did not observe such relationships between longitudinal changes in bone, lean, and fat tissue mass in 60 men with HF who were followed up for between 2 and 4 years. A reduction in bone mass was accompanied by an increase in fat mass (arms, legs, and total body), while no changes were found in lean mass (arms, legs, and total body).^33^ The authors hypothesized that bone tissue was the body compartment where wasting due to anabolic depletion occurs first, which is in line with the assumption that fat is usually lost later in the catabolic process of HF than muscle.^36^ Reduced BMD with subsequently increased susceptibility to fractures has been noted in patients after heart transplantation, but in this group, increased bone loss is primarily mediated by high doses of glucocorticoids and cyclosporin therapy.^37^

**Table 1:** Systematic review of studies on bone status in heart failure

Author	Journal Year	No. of subjects	Study population	Main findings		
				BMD	Bone markers	Other
Bozic *et al*.^32^	*J Card Fail* 2009	93 (73 with HF)	Men stable EF <40%	↓BMD in HF vs. controls BMD determined by serum adiponectin	↑β‐CTx, OC, OPG, RANKL in HF	—
Jankowska *et al*.^33^	*Eur J Heart Fail* 2012	208 (187 with HF)	Men, mean age 60	↓BMD in HF vs. non‐HF BMD determined by HF severity	β‐CTx, OC were not predictors of bone loss	Significant bone loss in 35% of pts with HF was determined by ↑NYHA and ↓testosterone
Kenny *et al*.^34^	*Osteoporos Int* 2006	83 (60 with HF)	43 HF men, mean age 77 + 17 HF women, mean age 78	↓BMD in HF vs. controls BMD determined by HF severity, frailty status, and vitamin D	—	—
Terrovitis *et al*.^39^	*Eur J Heart Fail* 2012	73 (60 with HF)	Men, mean age 57	↓BMD in HF vs. non‐HF ↓BMD in NYHA III/IV vs. I/II	—	↓BMD associated with impaired prognosis
Majumdar *et al*.^48^	*J Clin Endocrinol Metab* 2012	49 509 (1841 with HF)	Subjects >50 years old	↓BMD in HF vs. non‐HF	—	30% increased risk of osteoporotic fractures in HF
Wu *et al*.^49^	*Eur J Heart Fail* 2012	102 (88 with HF)	34 pts with VAD implantation	—	↑β‐CTx and NTX, =OC, P1NP in HF	VAD implantation: ↑P1NP, ↓β‐CTx, and NTX
Zotos *et al*.^50^	*J Osteopor Phys Act* 2014	73 (60 with HF)	Men, mean age 56 EF <40%	—	↑β‐CTx in HF ↑β‐CTx related with HF severity ↑β‐CTx predictor of adverse clinical events	—
Schleithoff *et al*.^51^	*J Bone Min Metab* 2003	42 (21 with HF)	Men, mean age 66 EF 29%	—	↓OC, =β‐CTx, NTx	No biochemical signs of enhanced bone resorption in HF men
Chen *et al*.^59^	*PLoS ONE* 2012	115	Pts with stable HF (EF <45%), mean age 59	↓Hip BMD in NYHA III vs. NYHA II	↑OPG in NYHA III vs. NYHA II	↑OPG associated with ↓hip BMD
Youn *et al*.^63^	*Osteoporos Int* 2015	65 with ADHF	50 men, mean age 60 15 women, mean age 69	40% osteoporosis and 53% vertebral fracture in women 12% osteoporosis, 32% osteopenia, 12% vertebral fractures in men	—	Exercise capacity predictor of bone mass

ADHF, acute decompensated heart failure; BMD, bone mineral density; β‐CTx, β‐CrossLaps; EF, ejection fraction; HF, heart failure; NTX, N‐telopeptides of type I collagen; NYHA, New York Heart Association; OC, osteocalcin; OPG, osteoprotegerin; P1NP, procollagen‐1 N‐terminal peptide; Pts, patients; RANKL, receptor activator of nuclear factor‐κB ligand; VAD, ventricular assist device.

### Impaired prognosis of patients with both heart failure and osteoporosis

Osteoporosis may have substantial prognostic significance in HF.^38^ Among patients with HF, lower BMD was associated with increased rates of death, implantation of an LV assist device, and/or inotrope dependency.^39^ When HF and osteoporosis are both present in a patient, subsequent mortality is more than additive.^40^ In men and women with HF, those with both HF and hip fractures have a two‐fold increased mortality relative to persons with HF without hip fractures.^40^ HF is also a major risk factor for mortality following hip fracture.^41^, ^42^

### Hip fracture as a fatal complication of osteoporosis in heart failure

The importance of low BMD and deteriorated bone metabolism in patients with HF is in the increasing risk of fractures. This is compounded with poor physical performance, which will increase the risk of falls. In general, HF is associated with a four‐fold higher risk of sustaining any fracture requiring hospitalization compared with patients with other cardiovascular diagnoses.^43^ In addition, the fracture incidence was 6% within 12 months among stable and non‐cachectic HF patients.^44^ The most devastating complication of osteoporosis is hip fracture, which is associated with a high mortality risk and among those who survive, can lead to a loss of function and independence often necessitating admission to long‐term care.^45^ In the Cardiovascular Health Study, a trend for a relationship between incident HF and incident hip fractures was reported.^40^ Similarly, in the Rochester Epidemiology Project, both prevalent and incident osteoporotic fractures were more common in HF patients compared with age‐matched and sex‐matched community controls, and this increased risk was almost entirely attributable to hip fractures.^46^ Osteoporotic fracture sites other than the hip have also been related with HF.^47^ In one study, approximately one‐tenth of patients with HF were found to have vertebral compression fractures on radiographs, one half of these had multiple fractures, while the majority (85%) was not on treatment for osteoporosis.^47^ In a population‐based study in Manitoba, Canada, including over 45 500 adults with 2703 incident osteoporotic fractures, which linked BMD data from 1998 to 2009 in those aged over 50 years with administrative databases, there was a 30% increase in major fractures in persons with HF.^48^ Instead of measuring bone mass directly, the assessment of bone metabolism, which reflects ongoing bone remodelling, may be an alternative for identifying HF patients at risk of osteoporosis and taking preventive and therapeutic measures in time.

## Bone metabolism in heart failure measured by bone markers

Bone markers reflect early changes in bone metabolism and can thus provide insights in the physiology and pathophysiology of bone. Serum or urine levels outside of the normal range may be indicative for disturbances in bone turnover. Moreover, the assessment of bone turnover may reflect ongoing bone remodelling more accurately than bone mass measurements. They may be helpful in the prediction of future bone loss.^35^ Previous studies have reported conflicting results on collagen markers of bone metabolism in HF patients.^49^

### Standard markers of bone metabolism in heart failure

In adults, bone is constantly being remodelled, first being broken down (bone resorption) and then being rebuilt (bone formation). The resorption and reformation of bone are important for repair of microfractures and to allow modification of structure in response to stress and other biomechanical forces. Bone formation is normally tightly coupled to bone resorption, so that bone mass does not change. Bone diseases occur when formation and resorption are uncoupled. Several markers are available that measure bone turnover. These markers include collagen breakdown products and other molecules released from osteoclasts and osteoblasts during the process of bone resorption and formation. Osteocalcin and N‐terminal propeptide of type I procollagen (PINP) are markers specific to bone formation, while markers specific to bone resorption include β‐CrossLaps (β‐CTx). We have previously described increased serum levels of osteocalcin and β‐CTx in HF patients indicating high bone turnover.^32^ Another study showed a strong elevation of bone resorption markers in HF patients that correlated negatively with LV ejection fraction.^44^ Zotos *et al*. have shown that β‐CTx is correlated with the severity of HF and with the decrease in BMD.^50^ These bone markers, which accurately reflect early changes in bone metabolism, can be used to predict future bone loss. In addition, the osteoclastic marker β‐CTx was strongly correlated with high morbidity and mortality in HF patients. To the best of our knowledge, it is the first marker of bone metabolism identified as a prognostic factor in chronic HF. Serum β‐CTx can be easily measured and may be an interesting addition to an overall assessment of chronic HF, as a prognostic tool and for the early identification of patients at risk of osteoporosis. However, another study reported no significant differences in bone resorption (measured by β‐CTx levels) in advanced HF.^51^ Less is known regarding bone formation markers and metabolism in patients with HF. However, Wu *et al*. did not find lower PINP levels in patients with advanced HF.^49^ PINP reflects an early phase of bone formation, namely, synthesis of procollagen type 1. Later stages of bone formation that reflect mineralization of previously synthesized type 1 collagen are reflected by osteocalcin. Wu *et al*. did not find differences in osteocalcin in advanced HF patients. Similarly, Jankowska *et al*. showed that none of the investigated bone metabolism markers (osteocalcin and β‐CTx) predicted bone loss during follow‐up in men with chronic HF.^33^ Some discrepancies in the available studies may be explained by differences in populations under study.

### The role of receptor activator of nuclear factor‐κB ligand and osteoprotegerin in bone metabolism in patients with heart failure

**Figure 1:**
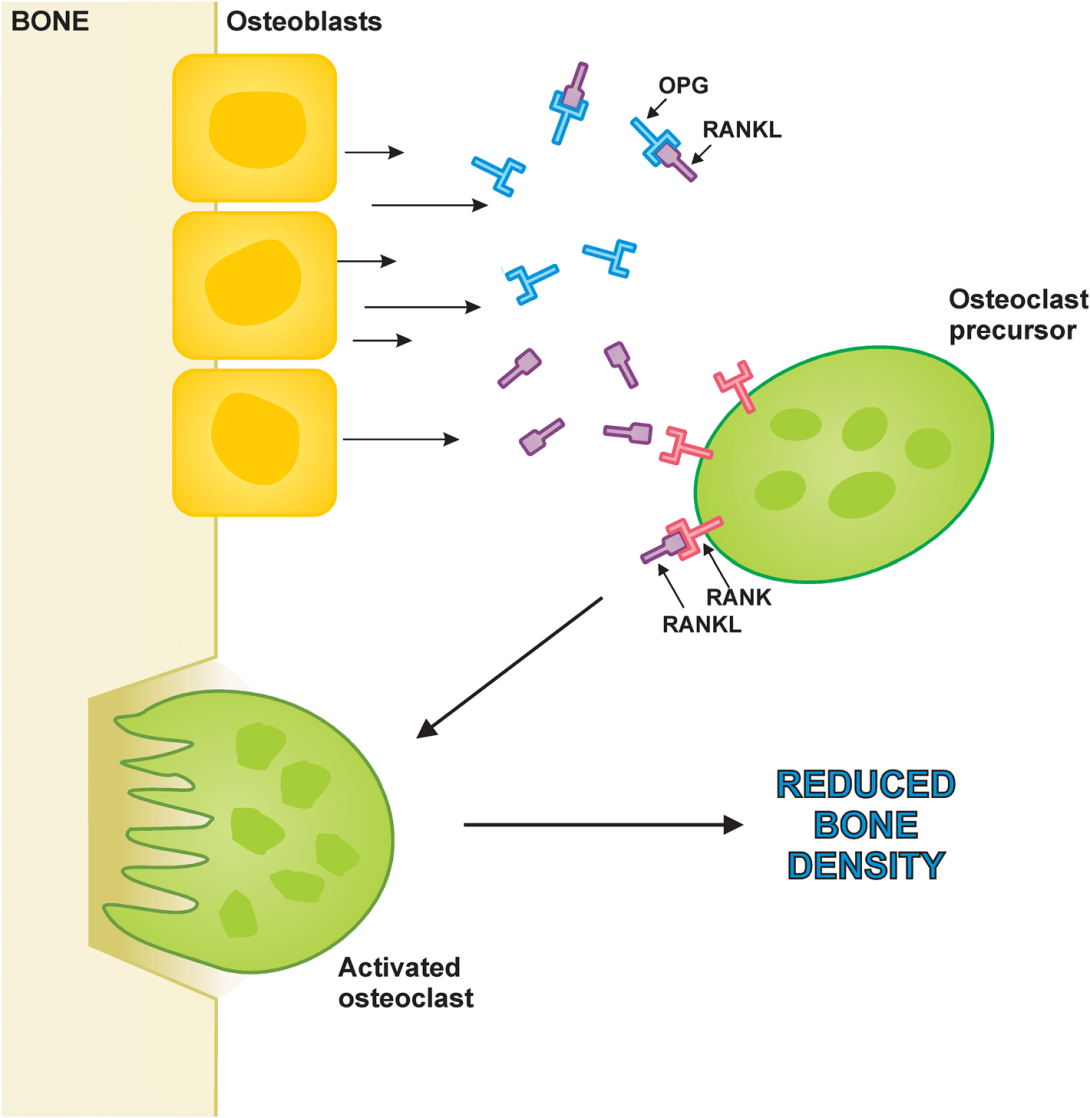
RANKL/osteoprotegerin interaction at the level of the bone. The essential signalling pathway for normal osteoclastogenesis. Under physiologic conditions, RANKL produced by osteoblasts binds to RANK on the surface of osteoclast precursors, triggering the transcription of osteoclastogenic genes. OPG inhibits the initiation of the process by binding to RANKL. OPG, osteoprotegerin; RANKL, receptor activator of nuclear factor‐κB ligand.

The threshold level for bone loss or bone formation can be influenced by various local and systemic factors, which are known to modulate the bone remodelling process. Nuclear factor‐κB is an important transcription factor in many cells types whose activation usually yields catabolic signalling. Several local factors such as receptor activator of nuclear factor‐κB ligand (RANKL) and osteoprotegerin (OPG) originate from osteoblasts/stromal cells (*Figure* 1). They play a pivotal role in the control of osteoclastogenesis. RANKL is a key link between reduced oestrogen levels and osteoclast‐mediated bone loss in post‐menopausal women.^52^, ^53^, ^54^ Briefly, RANKL is involved in the differentiation and activation of osteoclasts, while OPG is associated with osteoclasts inhibition and apoptosis.^55^ Data from transgenic animals have shown that overexpression of RANKL can result in osteoporosis. OPG administration is able to reduce bone resorption processes in post‐menopausal women.^55^ By binding RANKL and acting as a decoy receptor to competitively inhibit RANKL interaction with its receptor, OPG subsequently inhibits the production and differentiation of osteoclasts. As a result, bone resorption is inhibited.^56^ We have previously demonstrated an elevated OPG in chronic HF patients.^32^, ^57^ The cause of high OPG in HF may be deduced through similar observation in natriuretic peptides in HF. In the GISSI‐HF trial, OPG did also carry a prognostic function in patients with chronic HF.^58^ Chen *et al*. have noted an inverse relationship between OPG and BMD in HF patients.^59^ They suggested that OPG may be used as an indicator of osteopenia or osteoporosis clinically. However, the mechanism underlying OPG and BMD interaction in HF warrants further randomized prospective, outcome studies in larger population as well as basic research efforts.

## Pathophysiology of reduced bone mass in heart failure

**Figure 2:**
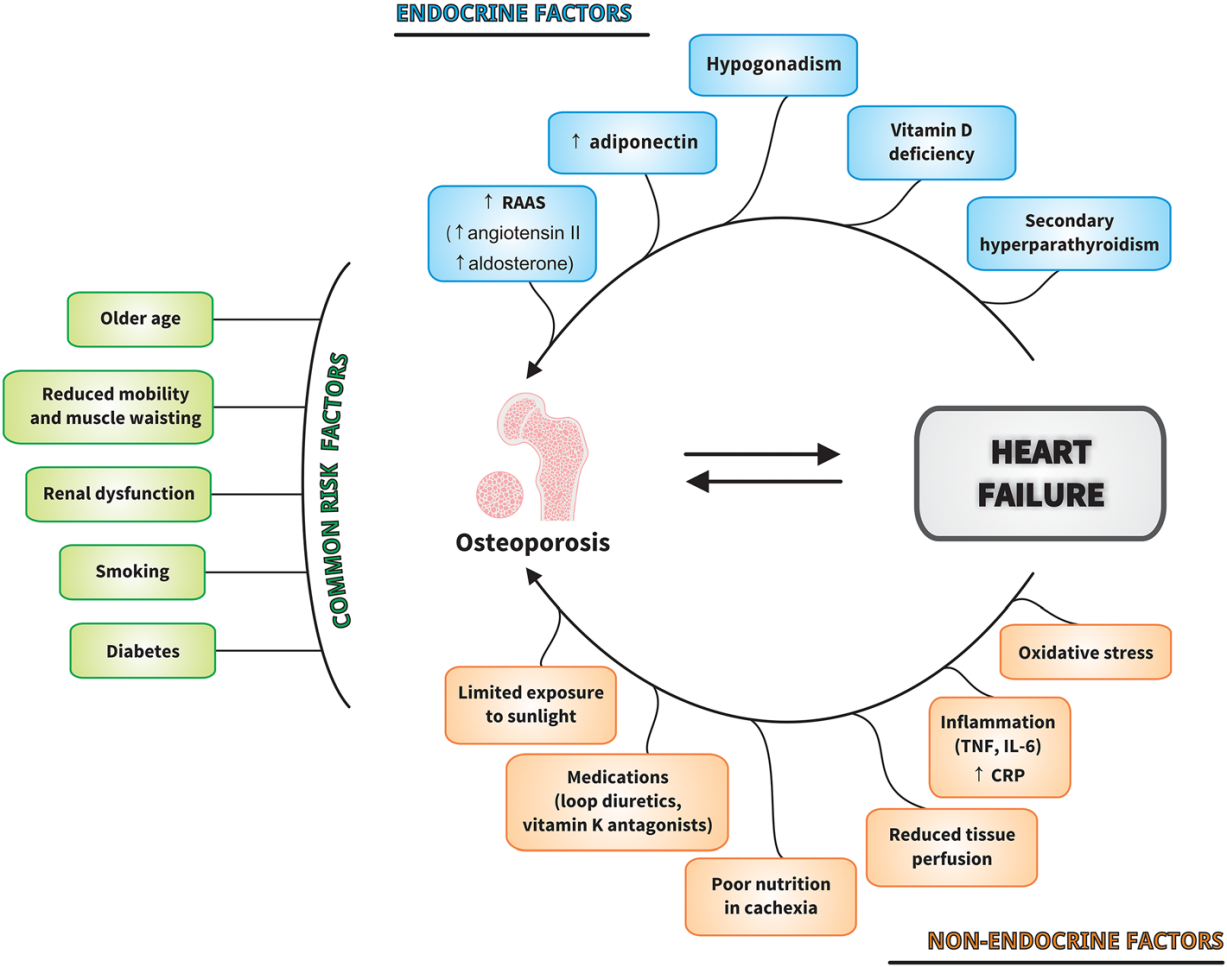
Factors contributing to bone loss in heart failure. CRP, C‐reactive protein; IL‐6, interleukin‐6; RAAS, renin–angiotensin–aldosterone system; TNF, tumour necrosis factor.

The pathophysiology of reduced bone mass in HF is not well understood. Aging is the most prominent confounder in the development of both bone loss and HF. Aging is a complex process that ultimately leads to morbidity including cardiovascular diseases and changes in body composition. The prevalence of HF rises to ≥10% among people over 70 years of age.^1^, ^17^ Similarly, with aging, many changes in body composition occur including decrease of lean mass and BMD.^60^ As a result of aging of the human population worldwide, both HF and osteoporosis represent increasing burden for health care systems. With the aging of the human population, there is increasing risk of the diseases of the elderly including dementia. The burden of mortality from HF with dementia in the USA has been recently pointed out in the elderly population.^61^ On the other hand, the presence of dementia increased importantly the mortality risk after hip fracture surgery.^62^ More than one‐third of people with dementia will die after hip fracture surgery in a 1 year follow‐up and about one in two in more than 1 year follow‐up. Patients with dementia usually have less activity and poor self‐care ability leading to bone loss. Thus, the presence of dementia should be considered as the risk factor for bone loss, falls, and hip fracture in elderly patients with HF. Many non‐endocrine and endocrine factors, which are firmly interrelated, may contribute to bone loss in HF (*Figure* 2). In addition to decreased physical performance in HF and sharing a number of common risk factors such as renal insufficiency, smoking, and diabetes, accelerated bone loss may also come from vitamin D deficiency and hyperparathyroidism, elevated aldosterone levels, and loop diuretic use in subjects with HF.^63^, ^64^, ^65^

### Reduced mobility and muscle wasting

Patients with advanced HF have reduced mobility and decreased outdoor physical activity, which could influence vitamin D levels and bone metabolism.^38^ Exercise capacity independently predicts lumbar BMD, hip BMD, and femoral cortical thickness even after the adjustment for confounding variables.^66^ Muscle forces cause the largest burden on bones and the largest bone strains. Skeletal unloading due to prolonged bed rest or poor exercise capacity in HF patients leads to bone loss via reductions in the mechanical forces applied to bones.^67^ The mechanism of this bone loss appears to be a consequence of a decrease in the bone formation rate and a concurrent increase in the bone resorption rate.^68^ Skeletal muscle mass is an important predictor of exercise capacity, and it is an independent prognostic indicator of survival in HF patients.^6^, ^69^, ^70^ Some degree of muscle wasting is common even in mild HF.^71^ According to the muscle hypothesis, changes in the skeletal musculature are at the core of the deterioration of patients with HF.^72^ A loss of leg muscle mass seems to be an early event in the natural course of non‐cachectic chronic HF.^71^, ^72^, ^73^

### Low vitamin D and secondary hyperparathyroidism

In HF, the increased losses of urinary calcium, due to long‐term use of diuretics, the impaired absorption of these minerals and of vitamin D due to intestinal congestion, the decreased synthesis of 1,25‐dihydroxyvitamin D secondary to liver congestion, renal insufficiency, and limited sunlight exposure, and finally, the high aldosterone levels are all causes of secondary hyperparathyroidism.^74^, ^75^ The presence of vitamin D deficiency in HF population was more prominent than in healthy groups, that is, 90% of HF patients were vitamin D deficient, even in sunny climates.^76^, ^77^, ^78^ Secondary hyperparathyroidism was highly prevalent in elderly men with HF and was associated with impaired long‐term survival.^76^ Additionally, HF patients with secondary hyperparathyroidism had more severe disease compared with those patients with normal serum parathyroid hormone (PTH) levels.^76^ Increased levels of PTH in patients with HF potentiate bone resorption and thus bone loss. Terrovitis *et al*. have shown that hyperparathyroidism was correlated with both severity of HF and decreased bone density, suggesting that increased PTH levels in HF might be an element of the underlying pathophysiological pathway leading patients to osteopenia or osteoporosis.^39^

### The role of the neurohumoral activation

There is a critical role for the renin–angiotensin–aldosterone system in both HF and osteoporosis.^79^, ^80^ The angiotensin II receptor is a cell surface G‐protein coupled receptor, which upon activation leads to intracellular increases in cyclic AMP.^81^ Activation of cyclic AMP signalling pathway can up‐regulate receptor activator of RANKL expression in osteoblasts and subsequently activate osteoclasts responsible for bone resorption.^81^, ^82^ In addition, angiotensin II regulates blood flow in both bone capillary and blood vessels' endothelium, providing a functional link between osteoporosis and atherosclerosis.^82^ In addition, neurohormonal activation, manifested by elevated aldosterone levels, has been demonstrated to play a crucial role in the risk of orthopaedic fracture in animal models.^64^ Furthermore, the use of spironolactone is inversely associated with fractures in men with HF, suggesting that aldosterone may be important in fracture risk.^83^ Enhanced sympathetic outflow is one of the most important detrimental neurohumoral activation in HF. The importance of this model became even clearer when it was discovered that blockade of this sympathetic overactivity by beta‐blockers prolongs survival in patients with HF.^84^ In animal studies, activation of the sympathetic nervous system led to the activation of β‐adrenergic receptors on bone‐forming osteoblasts, resulting in bone loss.^85^ Khosla *et al*. evaluated the role of sympathetic outflow and presented evidence to support the idea that the sympathetic nervous system regulates bone metabolism in humans, primarily via the β1‐adrenergic receptor.^86^ Overall, growing clinical evidence suggests that pharmacological β1‐adrenergic receptor‐selective blockade may deliver a small but significant increase in bone mass and thus aid in the prevention of fractures.^86^, ^87^ The use of beta‐blockers is recommended standard treatment in patients with HF. However, we have found neither the study on the effect of the enhanced sympathetic outflow on bone status nor the evaluation of potentially beneficial effect of β‐blockade on bone in the context of HF. Additionally, the significantly increased levels of cortisol in HF contribute to the predominance of catabolic factors.^16^ Reduced circulating levels of anabolic hormones and/or increased concentrations of catabolic hormones are correlated to reduced muscle mass, fat tissue, and bone mass.^73^ Excess of glucocorticoids (cortisol) directly inhibits bone forming cells and thus may contribute to the development of osteoporosis.^88^

### Adiponectin as a link between heart failure and bone loss

Adiponectin, an adipocytokine secreted by adipocytes, also plays a role in both cardiovascular disease and osteoporosis.^89^, ^90^ Elevated adiponectin levels in HF reflect an attempt by the body to compensate for the impaired metabolism in this disease.^89^ Relative to osteoporosis, adiponectin stimulates osteoblast proliferation and differentiation.^91^ Previous meta‐analysis including 51 studies found that adiponectin is the most relevant adipokine negatively associated with BMD independent of gender and menopausal status.^92^ In line with this finding, we have previously demonstrated that increased serum adiponectin levels were independent predictor of reduced BMD in elderly men with mild to moderate HF and showed a positive correlation to bone‐specific surrogates.^32^ Furthermore, higher adiponectin levels are associated with increased fracture risk independent of body composition and BMD.^90^

### Hypogonadism

In elderly men, in particular, in men with HF, hypogonadism is a prevalent condition.^93^, ^94^ Hypogonadism is a risk factor for osteoporosis, and testosterone replacement can increase BMD.^95^ Reduced BMD and BMC in men with HF were predicted by depleted gonadal and adrenal drive (as evidenced by low serum levels of testosterone and dehydroepiandrosterone sulphate) and thus contribute to general body wasting.^33^ However, we did not find an association between total testosterone and estimated free testosterone neither with bone status nor with lean mass and muscle strength.^96^ Literature data on androgen status in men with HF, assessed on the basis of serum levels of testosterone, are equivocal. In an unselected cohort of men with HF, Jankowska *et al*.^97^ have shown decreased serum levels of androgen hormones (testosterone and estimated free testosterone), which were markers of poor survival. In line with the results of Anker *et al*.^16^ we have found no difference in serum levels of testosterone between elderly men with HF and healthy controls.^96^ Additionally, androgen status was not predictive of long‐term survival in our cohort of HF patients without diabetes.^96^ Difference in patients populations may be explanation of discrepancy between study results. Unlike to our study, Jankowska *et al*.^97^ included younger patients with more impaired renal function, while almost one‐third of study patients were diabetics. In general, diabetes may be considered as the most prominent potential confounding variable that can lead to this discrepancy between studies.

### Oxidative stress and inflammatory cytokines

Oxidative stress is at least partly responsible for the development and progression of HF.^98^ Reactive oxygen species directly impair contractile function by modifying proteins central to excitation contraction coupling, activate hypertrophy signalling kinases and transcription factors and mediate apoptosis, stimulate fibroblast proliferation, and activate matrix metaloproteinase.^99^ An imbalance between oxidant and antioxidant statuses is associated with increased osteoclastic and decreased osteoblastic activity.^100^ Inflammatory cytokines may also alter the bone metabolism.^101^ Tumour necrosis factor and interleukin‐6 are increased in chronic HF, and inflammation might play an important role in its development and progression (‘cytokine hypothesis’).^102^, ^103^ Interleukin‐6 levels are increased in HF patients and correlated with markers of bone turnover. Therefore, in this inflammatory state, the resorption of bone may be promoted by pro‐osteoclastogenic cytokines.

### Reduced tissue perfusion

Factors related to HF may play a role in reduced bone mass. For example, reduced tissue perfusion in patients with HF might block the proliferation and differentiation of osteoblasts, while strongly stimulating osteoclasts.^66^ However, the resorptive function of osteoclasts is not disturbed under hypoxic conditions.^104^ The negative impact of HF on bone metabolism might be associated with the direct impact of reduced pO_2_ and pH.^104^

### Drugs

A number of drugs used to treat HF can impact the risk of developing osteoporosis. Loop diuretic use has been associated with increased loss in BMD at the hip in men, and prolonged use of loop diuretics has been directly associated with total fractures in post‐menopausal women, but not with hip or clinical vertebral fractures.^65^, ^105^ Vitamin K is required for the carboxylation of osteocalcin, and it has been shown that individuals supplemented with vitamin K had increased serum levels of carboxylated osteocalcin and decreased undercarboxylated osteocalcin.^106^ Therefore, vitamin K antagonists may have influenced the measurements of osteocalcin. Epidemiological studies show that this abnormal vitamin K metabolism may increase osteoporotic fracture risk.^107^ Initiation of antihypertensive drugs, including some standard drugs for the treatment of HF (angiotensin‐converting enzyme inhibitor, angiotensin II receptor blocker, or beta‐adrenergic blocker), is a risk factor for falls in the elderly.^108^ Current ESC guidelines on HF suggest that drug therapy in older patients should follow the recommendations but with special attention due to the frailty, co‐morbidities, cognitive impairment, and limited social support of this population.^17^ Thus, one of the recommendations is to optimize doses of HF medication slowly and with frequent monitoring of clinical status and to reduce polypharmacy (number, doses, and complexity of regime) in order to avoid side effects of drugs including orthostatic hypotension that predisposes to the falls and fractures in those patients.

## Treatment of bone loss in heart failure

The development of preventive and therapeutic strategies against bone loss, sarcopenia, and wasting disorders is perceived as an urgent need by health care professionals.^109^, ^110^, ^111^, ^112^ However, the treatment of bone loss and wasting leading to cachexia is an unresolved challenge to this day. As wasting is a multifactorial disorder, it is unlikely that any single agent will be completely effective in treating this condition; thus, it will be necessary to target different pathways. Additional research is warranted for further evaluation of the molecular pathways linking osteoporosis and HF, which could identify novel therapeutic targets for prevention of both conditions. Studies evaluating anti‐osteoporotic drugs exclusively in HF patients are missing.

### Exercise

Exercise appears to increase BMD. Greater physical activity is associated with a 9–17% increase in BMD during adolescence and young adulthood.^113^ Being a part of daily living, exercise is the easiest way to preserve and increase muscle mass; also, it is the most effective anabolic agent with many ancillary effects delivered at no or low costs. Muscle wasting might be present even in younger patients with HF, particularly in those who are in poor clinical condition.^7^ Exercise restores muscle stem cell mobilization, regenerative capacity, and muscle metabolic alterations via adiponectin/AdipoR1 activation in mice.^114^ Among all investigated therapeutic strategies, aerobic exercise training in HF patients is the best proven to counteract skeletal muscle wasting and is recommended by treatment guidelines for HF.^17^ Preservation of muscle mass and muscle strength is of crucial importance for bone health. Oscillatory whole‐body vibration is a novel exercise modality, which is performed on a vibrating platform that moves in sinusoidal oscillations and during which static and dynamic exercises can be performed.^115^ Whole‐body vibration therapy can provide a significant improvement in reducing bone loss and reduce risk of falls in post‐menopausal women.^116^ Furthermore, its application improved exercise capacity, lower limb performance, and quality of life in patients with chronic diseases such as chronic obstructive pulmonary disease and pulmonary arterial hypertension.^117^, ^118^ Thus, it would be of interest to assess the role of whole‐body vibration on body compartments as a feasible and easily accessible method of continuous and potentially home‐based physical exercise in patients with HF.

### Drugs and devices

At least 19 drugs that can regulate muscle mass have been reported in the literature so far.^119^, ^120^ These therapeutic interventions include use of anti‐inflammatory substances and appetite stimulants. It has been demonstrated that testosterone therapy reveals favourable effects in men with HF (e.g. improvement in exercise capacity, amelioration of neurohumoral activation, improvement in insulin sensitivity, and anti‐inflammatory effects).^121^, ^122^ Direct anabolic properties of testosterone or indirect effects of this therapy may also potentially improve bone homeostasis in HF patients. However, favourable effects of testosterone on bone mass have not been confirmed in all published studies.^123^ Recently, a study revealed that alendronate, one of bisphosphonates, may reduce the risk of HF compared with control subjects.^124^ However, other bisphosphonate users were at increased risk of HF.^124^ Therefore, further research on bisphosphonate and HF is warranted. To our knowledge, the efficacy/safety analysis of bisphosphonates in exclusively HF patients with osteoporosis has not been previously performed. Statins are drugs with seemingly multiple actions, for which a possible effect on bone mass has been suggested.^125^ However, such an effect is still discussed, and conflicting results have been published in this regard in the general population.^126^ No data on the effectiveness of these drugs exist in terms of effects on bone status in HF patients. Despite the publication of studies exploring various doses and forms of vitamin D supplementation in patients with chronic HF, there remains considerable uncertainty regarding the benefits of this therapeutic approach.^77^, ^78^, ^127^ In the majority of these studies, there was no effect of vitamin D supplementation on HF symptoms except in the study by Boxer *et al*.^128^ who observed an improvement in quality of life in HF patients allocated to vitamin D supplementation. However, even the most recent study did not find improvements in physical performance (6 min walk distance) in patients with HF after vitamin D supplementation, albeit beneficial effects on LV structure and function were noted.^129^ The single study that evaluated vitamin D supplementation on bone metabolism in patients with HF showed no influence on bone turnover markers.^127^ Wu *et al*. showed that the haemodynamic improvement and mechanical unloading after ventricular assist device implantation are associated with a trend towards lower serum PTH levels and lower bone resorption markers and significantly higher bone formation markers in HF patients.^49^ Improved renal function with increased estimated glomerular filtration rate would be expected to decrease PTH levels, therefore reducing the degree of secondary hyperparathyroidism.

## Gaps in evidence and future perspective

1. Major HF guidelines do not cover the screening method or patient education in terms of osteoporosis or osteoporotic fracture, even though HF represents a substantially increased risk of fracture. Future HF guidelines should consider this issue.
2. New data are required to prove that testosterone deficiency may be the mechanistic explanation for bone loss in men with HF. A desirable methodological approach here would be an assessment of whether correction of testosterone deficiency could improve bone mass in these patients.
3. Further research is needed to confirm the potential of adiponectin and other adipokines in the crosstalk between musculoskeletal system and energy metabolism in HF. Interventional studies using the application of adiponectin or its mimicking agent osmotin may provide new insight into whether there is a causal relationship between adiponectin and musculoskeletal depletion.
4. It would be interesting to assess the potential biochemical crosstalk between skeletal muscle and bone metabolism, via biohumoral markers produced by muscles called myokines, in both healthy subjects and patients with HF. The role of myokines may be of importance for beneficial influence of physical training in healthy subjects.^130^ However, there are no data evaluating if beneficial effect of physical training may be regulated by myokines in patients with HF.
5. The mechanism underlies OPG/RANKL, and bone status in HF warrants further randomized prospective, outcome study in larger population and bench works.
6. Secondary hyperparathyroidism is a potential cause for bone loss in the context of HF, which may be treated with cinacalcet. No study to date has evaluated the prognostic effect of adjustment of serum PTH by cinacalcet in HF patients. Cinacalcet prevents hyperparathyroidism and its consequences.^131^ Treatment with the drug significantly lowers the rates of cardiovascular death and major cardiovascular events in patients on haemodialysis.^132^ If future studies prove the beneficial prognostic effect of PTH adjustment, PTH may transform from risk marker to modifiable risk factor in the context of HF with establishment of new treatment target in this severe disease.
7. It would be of interest to assess efficacy/safety profile of anti‐osteoporotic drugs in the context of HF including bisphosphonates, vitamin D supplementation, selective oestrogen receptor modulator (raloxifene), and biologicals (denosumab).

## Conclusions

Heart failure and osteoporosis are highly prevalent aging‐related diseases that exact a huge impact on society. Published studies show that HF is related with reduced BMD and increased risk of osteoporotic fractures, especially in those with more severe HF. Both disorders are common causes of loss of function and independence, and of prolonged hospitalizations, presenting a heavy burden on the health care system. When HF and osteoporosis are both present in a patient, subsequent mortality is more than additive. On the other hand, current HF guidelines do not suggest screening methods or patient education for osteoporosis or osteoporotic fractures. Thus, this review and as well as other studies may serve as a base to discuss the necessities of bone health evaluation in HF patients.

## Funding

This work was supported by the European Commission's 7th Framework Programme (FP7, grant number 241558).

## Conflict of interest

Goran Loncar, Natasa Cvetinovic, Mitja Lainscak, Andjelka Isaković, and Stephan von Haehling declare that they have no conflict of interest. All authors declare that the submitted work has not been published before.
